# Aqueous, Unfolded OmpA Forms Amyloid-Like Fibrils upon Self-Association

**DOI:** 10.1371/journal.pone.0132301

**Published:** 2015-07-21

**Authors:** Emily J. Danoff, Karen G. Fleming

**Affiliations:** T. C. Jenkins Department of Biophysics, Johns Hopkins University, Baltimore, Maryland, United States of America; Aligarh Muslim University, INDIA

## Abstract

Unfolded outer membrane beta-barrel proteins have been shown to self-associate in the absence of lipid bilayers. We previously investigated the formation of high molecular weight species by OmpA, with both the transmembrane domain alone and the full-length protein, and discovered that the oligomeric form contains non-native β-sheet structure. We have further probed the conformation of self-associated OmpA by monitoring binding to Thioflavin T, a dye that is known to bind the cross-β a structure inherent in amyloid fibrils, and by observing the species by electron microscopy. The significant increase in fluorescence indicative of Thioflavin T binding and the appearance of fibrillar species by electron microscopy verify that the protein forms amyloid-like fibril structures upon oligomerization. These results are also consistent with our previous kinetic analysis of OmpA self-association that revealed a nucleated growth polymerization mechanism, which is frequently observed in amyloid formation. The discovery of OmpA’s ability to form amyloid-like fibrils provides a new model protein with which to study fibrillization, and implicates periplasmic chaperone proteins as capable of inhibiting fibril formation.

## Introduction

**Fig 1:**
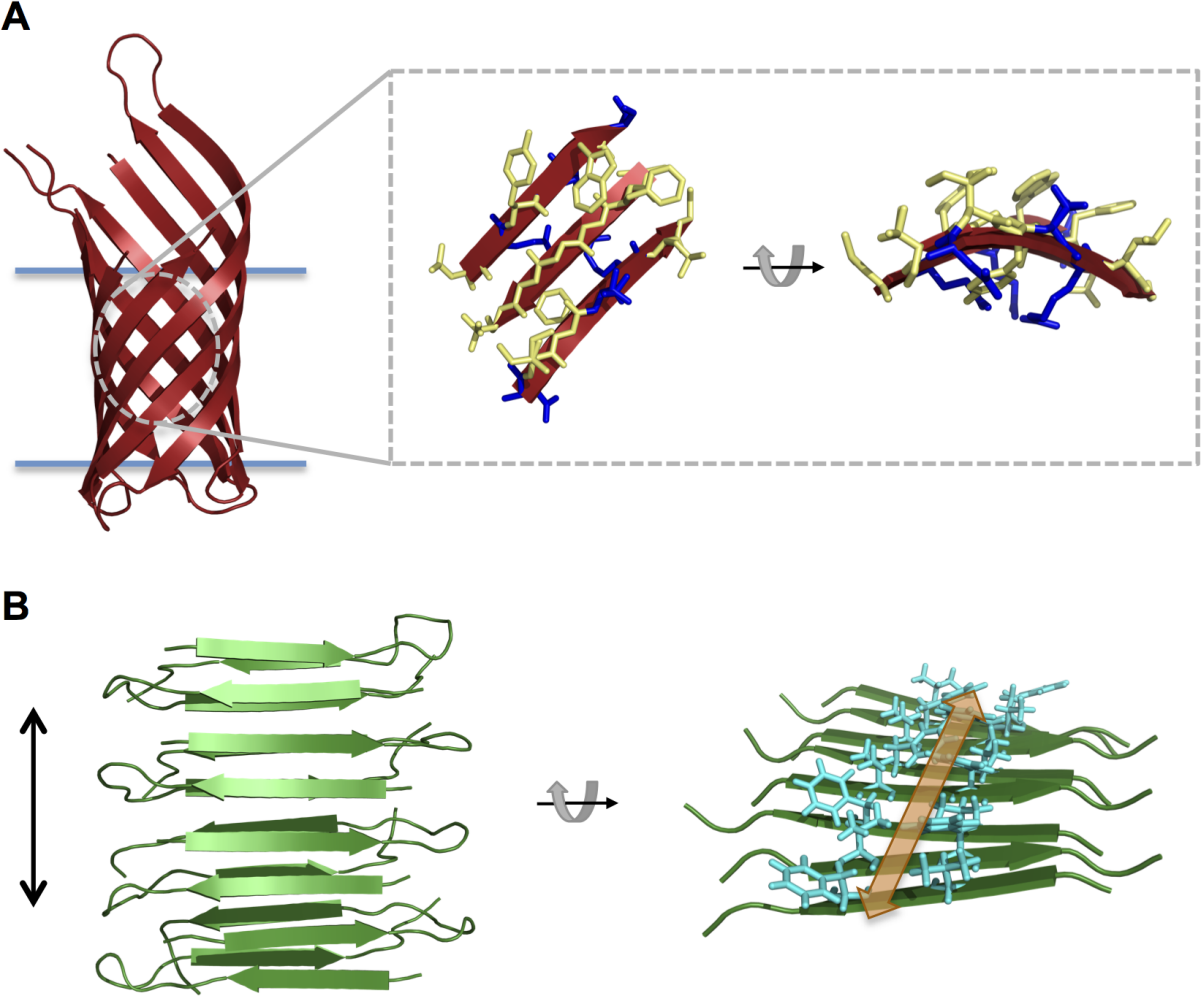
Illustration of protein structures. (A) Cartoon depiction of the transmembrane domain of OmpA (PDB id 1QJP [3]) with the membrane indicated by blue horizontal lines. A zoomed-in section of β-sheet is shown in stick representation with hydrophobic side chains depicted in yellow and hydrophilic side chains depicted in blue. A rotated side view of the β-sheet section is shown on the right. (B) Cartoon depiction of the cross-β structure of the amyloid-forming peptide D23N-Aß_1-40_ (PDB id 2LNQ [4]). The fibril axis is indicated by a vertical black arrow. A rotated view of the structure is shown on the right (only one β-sheet is shown for clarity) to illustrate the proposed binding groove for the dye Thioflavin T. Side chains lining the groove are shown as cyan sticks and the binding groove is indicated by an orange arrow.

In transmembrane proteins, the amino acid residues that reside in the membrane interior generally possess hydrophobic side chains in order to have an energetically favorable interaction with the nonpolar lipid acyl chains [1]. This also results in a high propensity for aggregation in the absence of the bilayer environment, especially for α-helical membrane proteins, which contain continuous stretches of hydrophobic residues; this also explains the common need for detergents when purifying these proteins. β-barrel outer membrane proteins (OMPs) offer the advantage of a lower aggregation propensity due to their sequence pattern of alternating hydrophobic and hydrophilic residues—a consequence of only every other residue in the transmembrane β-strands facing the lipid environment [2]. This structural arrangement is illustrated in Fig 1A for Outer Membrane Protein A (OmpA)[3]. The side chains are shown for a section of β-sheet from OmpA, with hydrophobic residues depicted in yellow and hydrophilic residues in blue. It can be seen that in each strand the side chains are oriented in alternating directions and the outward facing residues are predominantly hydrophobic while the inward facing residues are predominantly hydrophilic (particularly evident in the rotated side view on the right).

However, it has been shown that aqueous unfolded (U_AQ_) OMPs do self-associate to form non-native, high molecular weight species in low denaturant conditions, which compete with productive folding *in vitro* [5]. We previously investigated the aggregation propensities of eight OMPs from *Escherichia coli*, and observed varying degrees of U_AQ_ self-association as a function of urea concentration, pH, and ionic strength [6]. OmpA, which has frequently been used as a model protein to study the folding of OMP β-barrels, exhibited very little tendency to self-associate. In a subsequent study, we determined that the U_AQ_ transmembrane barrel domain of OmpA does form high molecular weight oligomers at lower denaturant concentrations (e.g. 600 mM urea), and as a function of protein concentration and time [7]. We also observed that the self-associated form of the protein gained β-sheet structure distinct from the native β-barrel fold, based on the circular dichroism (CD) spectra. In addition, analysis of the CD kinetics of oligomerization led to the conclusion that OmpA self-association follows a nucleated growth polymerization reaction with a critical nucleus size of three molecules [7]. Based on these observations, we hypothesized that the U_AQ_ OmpA barrel domain forms amyloid-like structures upon self-association, but further experimentation was needed to verify this.

Amyloid-forming proteins are associated with many human diseases, such as the neurodegenerative conditions of Alzheimer’s disease and Parkinson’s disease. Such protein deposition diseases involve the conversion of these proteins from their soluble states to long fibrillar aggregates, termed amyloid fibrils [8]. Although initially it was thought that fibrillar deposits were the causative agents of toxicity, more recent work has provided evidence that amyloid fibril precursors such as oligomers or protofibrils are the pathogenic species that lead to disease [8].

Amyloid fibrils are highly ordered protein aggregates that typically consist of several protofilaments twisted together. Within each protofilament, protein molecules are arranged in β-strands that run perpendicular to the fibril axis, a topology referred to as “cross-β” structure [8]. This configuration is illustrated in Fig 1B for the amyloid-forming peptide D23N-Aß_1-40_ [4]. The fibril axis is indicated by a vertical black arrow.

In addition to CD, which is a sensitive indicator of β-sheet secondary structure, two common techniques used to verify the presence of amyloid fibrils are specific binding to the dye Thioflavin T (ThT), which is dependent on the cross-β conformation, and transmission electron microscopy to assess aggregate ultrastructure. Here we describe the use of these techniques to verify amyloid fibril structure for the oligomeric form of U_AQ_ OmpA, supporting the previously observed β-structured CD spectrum and nucleated growth polymerization mechanism [7].

The formation of amyloid fibril structure by OmpA has important implications: it provides another protein class with which to study the process of fibrillization and the sequence properties contributing to amyloid formation, and raises the biological question of how periplasmic chaperone proteins prevent the formation of amyloid fibrils by client OMPs.

## Materials and Methods

### Preparation of OmpA_325_ and OmpA_171_

The cloning and expression of full-length OmpA (OmpA_325_) and the N-terminal barrel domain (OmpA_171_) have been described previously [7]. In brief, the gene sequences were PCR-amplified and ligated into a pET11a vector, and the proteins were expressed to inclusion bodies in hms174(DE3) cells with induction by 1 mM IPTG (Fisher). The proteins were purified in Urea Buffer (8 M urea, 20 mM Tris, pH 8) prepared with ultra-pure, deionized urea (Amresco), using anion exchange and gel filtration on a BioRad BioLogic Duoflow Chromatography System as described [7]. For OmpA_325_, Urea Buffer contained 2 mM TCEP (Pierce). Protein concentrations were determined by spectroscopy using previously calculated extinction coefficients [7]. Purified protein was stored in aliquots at -80°C until use.

### Circular dichroism

Circular dichroism (CD) measurements were conducted using an Aviv Circular Dichroism Spectrometer, Model 410 (Aviv Biomedical), with a custom inset detector to reduce the effects of light scattering. Samples were prepared of unfolded OmpA_171_ at a concentration of 1 μM in 600 mM urea (monomer conditions) or 10 μM in 600 mM urea (self-associating conditions). The self-associating sample was incubated for 16 h at 25°C before measurement. Natively folded OmpA_171_ was prepared by incubation for 5 h with large unilamellar vesicles (LUVs) at concentrations of 1 μM protein and 800 μM diC_10_PC (Avanti Polar Lipids) in 1 M urea. LUVs were prepared by reconstitution of dried lipid films in 1 M urea at a concentration of 10 mg ml^-1^ and extrusion through a 0.1 μm filter (21 passes) using a mini-extruder (Avanti) [7]. All samples were prepared in 20 mM Tris, pH 8.

CD wavelength spectra were recorded at 25°C between 200 and 280 nm in 1 nm increments, with an averaging time of 5 s. For each sample, three scans were recorded and averaged. Hellma cuvettes with a path length of 1 cm (monomeric and folded samples) or 1 mm (self-associated sample) were used and spectra of cuvettes containing only urea/buffer or LUVs/urea/buffer were subtracted from sample spectra to correct for background signal. Data were converted to mean residue ellipticity using the following equation:

$$[\Theta ]=\frac{\theta }{10cln}$$ (1)

where theta is the measured ellipticity in mdeg, *c* is the concentration in M, *l* is the path length in cm, and *n* is the number of residues (172, including the N-terminal methionine [7]).

### Fluorescence measurements of Thioflavin T binding

Thioflavin T (ThT) was purchased from Sigma and dissolved in Hydro ultra-pure water at a concentration of 500 μM. Samples were prepared of ThT alone or with OmpA_171_ or OmpA_325_ at concentrations of 3 μM ThT and either 1 μM protein in 600 mM urea (monomer conditions) or 3 μM protein in 300 mM urea (self-associating conditions). All samples were prepared in 20 mM Tris, pH 8. Self-associating samples were incubated for 16 h at 25°C before measurement.

Fluorescence measurements were conducted at 25°C with a 1 cm path length using an ISS PC1 spectrofluorometer. Excitation slits were 2.4 mm and emission slits were 2.0 mm. Excitation spectra were recorded from 350 to 480 nm with the emission monochromator set to 482 nm. Emission spectra were recorded from 460 to 600 nm with excitation at 450 nm. For each spectrum, three scans were recorded and averaged.

### Transmission electron microscopy

Samples were prepared of OmpA_171_ and OmpA_325_ under monomer conditions (1 μM protein in 600 mM urea) or self-associating conditions (3 μM protein in 300 mM urea or 10 μM protein in 800 mM urea). All mixtures were prepared in 20 mM Tris, pH 8. Self-associating samples were incubated for 16 h at 25°C. Negatively stained samples were prepared for transmission electron microscopy (TEM) using freshly ionized formvar/carbon-coated copper grids. The protein solutions were adsorbed to the grids for 5 minutes, then washed six times with water. The grids were stained with 2% uranyl acetate for 1 minute before removing excess stain. The samples were observed in an FEI Tecnai 12 TWIN electron microscope operating at 100 kV. Images were captured using an SIS Megaview III camera (Olympus). Width measurements of observed structures were made with ImageJ.

## Results

### Self-associated OmpA exhibits non-native β-sheet structure

**Fig 2:**
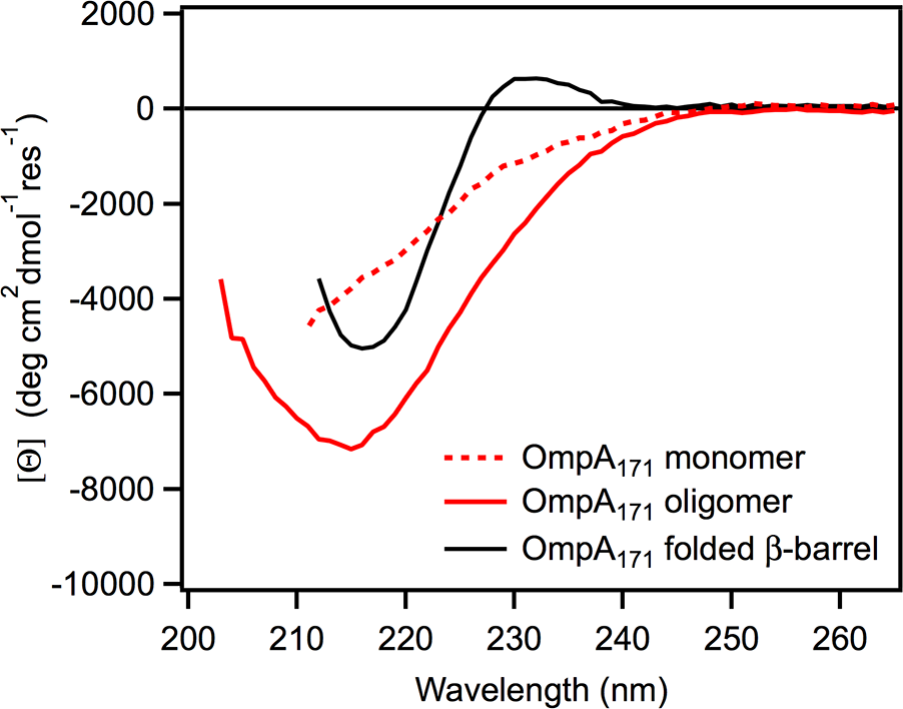
CD wavelength spectra of unfolded monomeric, oligomeric, and folded OmpA_171_. Monomeric OmpA_171_ (dotted red) was prepared at a concentration of 1 μM in 600 mM urea, 20 mM Tris, pH 8. Oligomeric OmpA_171_ (solid red) was prepared at a concentration of 10 μM in 600 mM urea, 20 mM Tris, pH 8 and incubated for 16 h. Natively folded OmpA_171_ (solid black) was formed by incubation with 800 μM diC_10_PC LUVs at 1 μM protein in 1 M urea, 20 mM Tris, pH 8.

We previously investigated the propensity of U_AQ_ OmpA to self-associate in the presence of low urea concentrations [7]. CD measurements revealed that the oligomeric form of the protein exhibited a spectrum indicative of β-sheet structure but with a different shape and intensity than the native β-barrel spectrum. Fig 2 shows the CD spectrum obtained for oligomeric OmpA_171_ (the N-terminal a-barrel domain of OmpA alone; solid red line)[7]. The protein was prepared at a concentration of 10 μM in 600 mM urea, 20 mM Tris, pH 8 and incubated for 16 h, a condition shown to result in the formation of high molecular weight oligomers [7]. Also plotted are the spectra for the unfolded, monomeric form of the protein (obtained with 1 μM protein in 600 mM urea; dotted red line) and the native a-barrel (1 μM protein folded into 800 μm diC_10_PC LUVs; solid black line).

It is evident that the monomeric protein has no regular structure while the oligomeric and folded forms exhibit troughs around 216 nm, indicative of β-sheet structure [9]. However, the trough for self-associated OmpA_171_ is more intense and broader, and the spectrum lacks the positive peak at 230 nm present in the native spectrum. A similar peak has been observed for the β-barrel OMP PagP and was attributed to a Cotton effect between aromatic groups formed only in the native barrel conformation [10]. As OmpA_171_ also contains a number of aromatic residues, we have proposed that a similar interaction occurs in this protein to give rise to the peak at 230 nm [7]. The absence of the 230 nm peak in the oligomeric OmpA_171_ spectrum indicates that the protein is not forming the native β-barrel in the absence of lipid bilayers. Conversely, the greater intensity of the a-trough in the oligomeric spectrum indicates that the self-associated form of OmpA_171_ contains a higher degree of a-sheet structure than the native barrel.

We also previously showed that the full-length OmpA protein (OmpA_325_), which contains a soluble periplasmic domain, exhibits a broad, intense β-sheet spectrum upon self-association as well [7]. Because the spectrum overlaid well with the mathematical sum of the spectra for oligomeric OmpA_171_ and the folded periplasmic domain, we concluded that the two domains are separate folding units and that the U_AQ_ barrel domain still self-associates when the periplasmic domain is present. However, we observed that self-association is slower for the full-length protein, indicating that the periplasmic domain has a slight chaperone ability to reduce the oligomerization rate of the barrel domain [7].

The presence of non-native β-sheet structure in self-associated OmpA_171_ and OmpA_325_ suggests the possibility that the proteins are forming amyloid-like structures, so we investigated this further using Thioflavin T and electron microscopy.

### OmpA oligomers bind Thioflavin T, indicating cross-β structure

Monitoring the fluorescence properties of the benzothiazole dye ThT has become an established method to detect amyloid fibrils, for both *in vivo* staining and *in vitro* quantification [11–14]. ThT has been shown to selectively bind amyloid fibrils, resulting in a novel excitation band ~450 nm and a large increase in fluorescence at 482 nm [12, 13], likely due to a loss in rotational freedom upon binding [15]. Although the interactions between ThT and amyloid proteins are not fully elucidated at the atomic level, it is thought that the dye binds in the grooves running parallel to the fibril axis, created by the repeating pattern of adjacent side chains in the stacked β-strands [15]. Fig 1B illustrates this groove in the amyloid fibril conformation of D23N-Aß_1-40_ [4]. In the rotated view of the β-sheet on the right, the side chains forming the groove are shown in stick representation and the area where ThT is proposed to bind is indicated by a double-headed orange arrow. Thus, the cross-β architecture common to all amyloid proteins is the basis of ThT binding.

**Fig 3:**
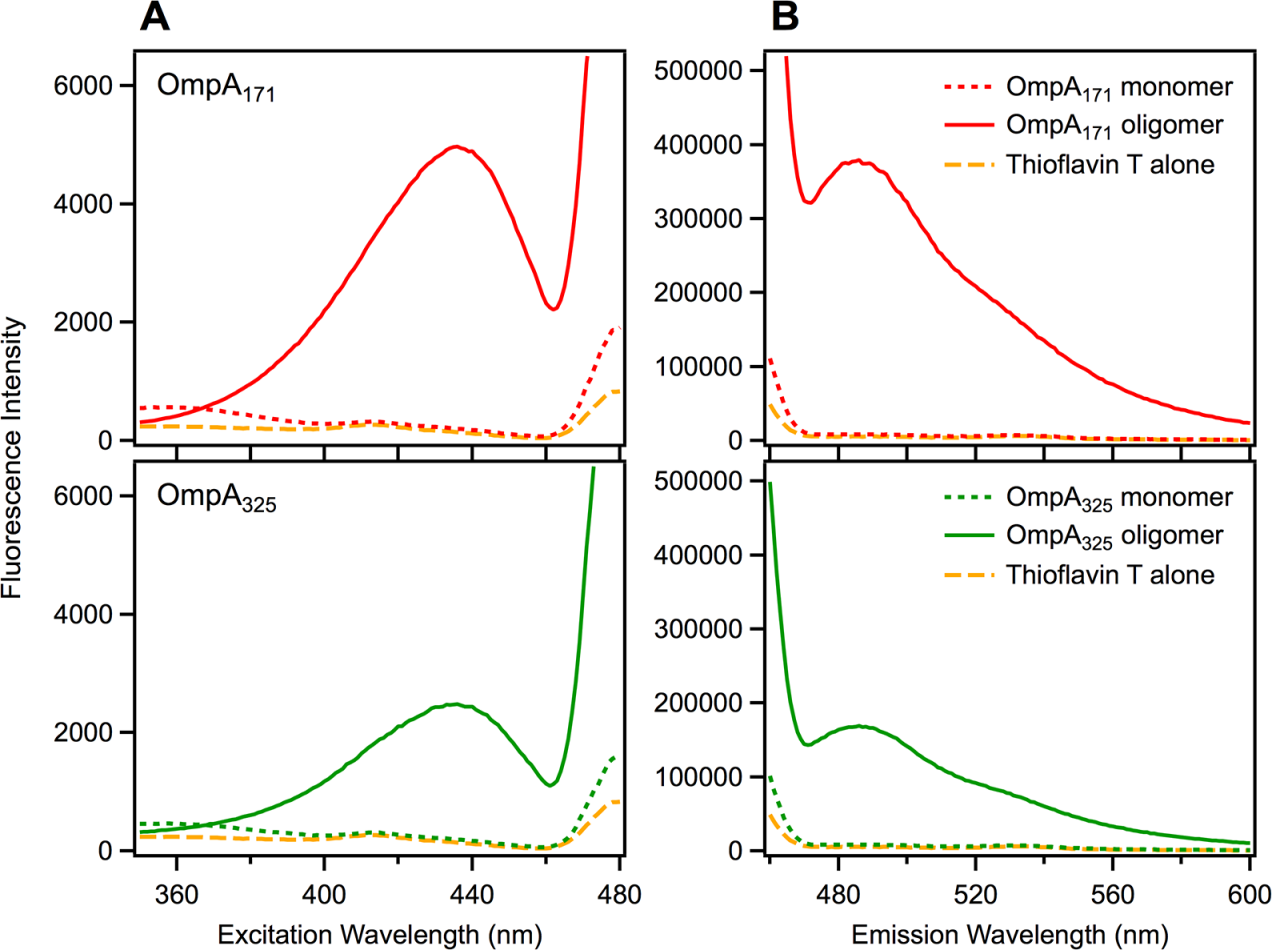
Thioflavin T binding measured by fluorescence. (A) Excitation spectra for 3 μM Thioflavin T in the presence of OmpA_171_ (top, red) or OmpA_325_ (bottom, green). Dotted lines correspond to conditions that maintain the monomeric state for both constructs (1 μM protein in 600 mM urea, 20 mM Tris, pH 8). Solid lines correspond to conditions that promote self-association for both constructs (3 μM protein in 300 mM urea, 20 mM Tris, pH 8, with a 16 h incubation). The spectrum for Thioflavin T alone is shown as a dashed gold line. Emission was collected at 482 nm. (B) Emission spectra for the same mixtures, with excitation at 450 nm.

To determine if the β-structured OmpA oligomers bind ThT, we prepared mixtures of the protein and dye under self-associating and monomeric conditions and measured fluorescence excitation and emission spectra. Fig 3A shows excitation spectra for OmpA_171_ (top, red) and OmpA_325_ (bottom, green) in the presence of 3 μM ThT (emission collected at 482 nm). The solid lines correspond to a self-associating condition for both constructs as determined previously (3 μM protein, 300 mM urea, 16 h incubation)[7] while the dotted lines correspond to a condition where both proteins remain entirely monomeric (1 μM protein, 600 mM urea, measured immediately). It is clear that the monomeric condition gives excitation spectra similar to the spectrum for ThT alone (dashed gold line) while the self-associating condition induces an excitation peak ~440 nm for both protein constructs. Fig 3B shows the emission spectra for the same conditions (excited at 450 nm) and it is evident that the self-associating condition exhibits a large emission peak at 482 nm for both protein constructs that is not seen under the monomeric condition or for dye alone. We therefore conclude that the oligomeric form of the U_AQ_ OmpA barrel domain does bind ThT and have a cross-β, amyloid-like structure.

Interestingly, both the excitation and emission peaks for oligomeric OmpA_171_ (top) are larger than for oligomeric OmpA_325_ (bottom), indicating a higher quantity of cross-β structure. This is consistent with the previous observation that U_AQ_ OmpA_325_ self-associates more slowly than OmpA_171_ due to the presence of the periplasmic domain [7].

### Electron microscopy of OmpA reveals fibrillar and spherical structures

**Fig 4:**
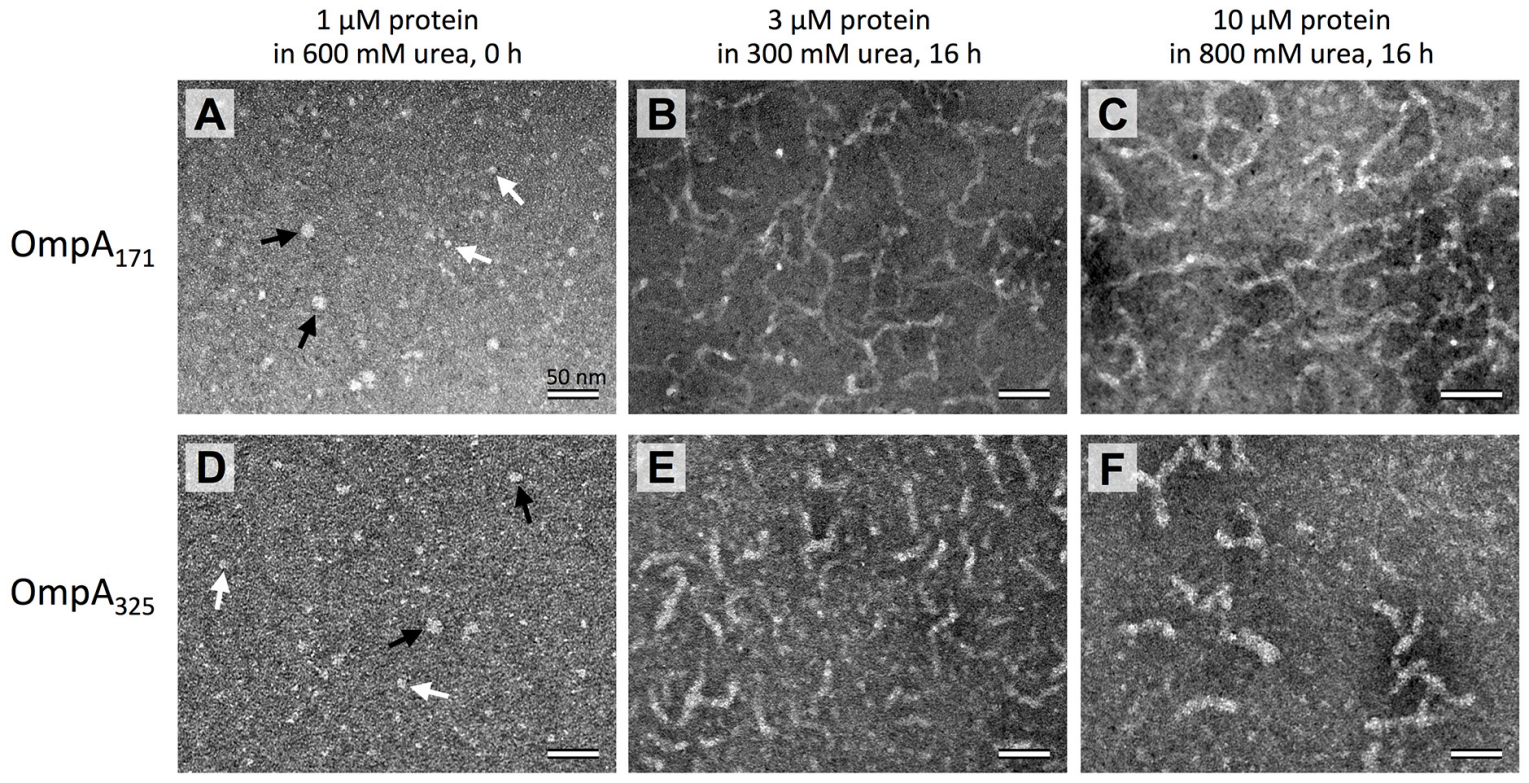
Transmission electron microscopy of OmpA. (A-C) OmpA_171_ prepared under monomeric (A; 1 μM protein in 600 mM urea), and self-associating (B; 3 μM protein in 300 mM urea, C; 10 μM protein in 800 mM urea) conditions. Self-associating samples were incubated for 16 h before measurements were performed. (D-F) OmpA_325_ under the same respective conditions as in (A-C). All samples were adsorbed to formvar/carbon-coated grids, negatively stained, and observed with an FEI Tecnai 12 electron microscope. In (A) and (D), large spherical objects are indicated by black arrows and small spherical objects are indicated by white arrows. Scale bars in all panels represent 50 nm.

To assess the ultrastructure of self-associated OmpA, we examined the protein by transmission electron microscopy (TEM). Fig 4 shows representative images of OmpA_171_ and OmpA_325_ under monomer conditions (1 μM protein in 600 mM urea) and two different self-associating conditions: 3 μM protein in 300 mM urea or 10 μM protein in 800 mM urea (corresponding to the self-associating conditions utilized in Fig 3 and Fig 2, respectively), both incubated for 16 h. Panels B and C show that OmpA_171_ forms long, flexible fibrils upon oligomerization. Although typical amyloid fibrils are long and rigid, similarly flexible fibrils have been observed for the amyloidogenic protein β-synuclein under certain conditions [16]. The fibrils formed by OmpA_325_ (panels E and F) are noticeably shorter than those for OmpA_171_. Again, this is consistent with U_AQ_ OmpA_325_ self-associating more slowly than OmpA_171_, resulting in less elongated fibrils in the same amount of time. In support of this, we also observed shorter-length fibrils for OmpA_171_ when the samples were incubated for 7 h instead of 16 h (data not shown).

In addition, the OmpA_325_ fibrils are slightly thicker than the OmpA_171_ fibrils: the fibrils formed by OmpA_171_ (panels B and C) have a measured thickness of 6–7 nm whereas the fibrils formed by OmpA_325_ (panels E and F) have a thickness of 10–12 nm. This could be due to the presence of the periplasmic domain in OmpA_325_. Being a globular domain, the periplasmic domains likely do not participate in the β-sheet stacking of the transmembrane domains, and instead probably extend out into the surrounding space, thus resulting in an overall greater fibril width. Still, the fibril thicknesses observed for both OmpA_171_ and OmpA_325_ are consistent with the typical amyloid fibril width of 7–13 nm [8, 16–18].

Fig 4A and 4D show TEM images of the proteins under monomeric conditions, and it is apparent that no fibrils are formed for either protein construct. However, large and small spherical objects are observed. For both OmpA_171_ and OmpA_325_, the larger spheres (indicated by black arrows) have a measured width of 11–12 nm and the smaller spheres (indicated by white arrows) have a width of 6–7 nm. It’s possible that the smaller spheres correspond to the monomeric form of the protein, but we hypothesize that the larger spheres are protein oligomers that correspond to protofibrils, precursor oligomeric species that subsequently assemble into larger fibrils. Protofibrils with spherical or annular structures have been observed previously for many amyloid fibril-forming proteins [19–23].

The appearance of spherical protofibrils under this condition was unexpected, because there was no observation of self-association for OmpA_171_ or OmpA_325_ under the same condition by sedimentation velocity analytical ultracentrifugation [7], nor was there any indication of cross-β structure by CD (Fig 2) or Thioflavin T binding (Fig 3)(although the latter could simply be evidence that the protofibrils lack regular β-sheet structure). One possible explanation for these observations is that the protein truly is monomeric under this buffer condition, but protofibril formation is induced through interaction with the TEM grid. This idea is supported by the observation that the larger spherical objects become more numerous when the same sample is adsorbed to the grid for 10 minutes instead of 5 minutes before washing (data not shown). Nonetheless, even if the formation of protofibril oligomers is induced by the TEM grid, the structures could still be biologically relevant on-pathway species for fibril formation, and thus their formation could provide an opportunity for further study of protofibril species.

## Discussion

We have shown that the U_AQ_ transmembrane domain of OmpA self-associates in low denaturant conditions lacking lipid bilayers to form high molecular weight species that exhibit substantial β-sheet structure, are capable of binding Thioflavin T, and appear as long flexible fibrils by TEM, thus leading to the conclusion that U_AQ_ OmpA forms amyloid-like fibrils. It is likely that other OMPs also form such structures, as high molecular weight species have also been observed for a number of OMPs in the absence of membranes [6]. This discovery has important implications for the amyloid field because it provides an entirely new class of proteins with which to study the process of fibrillization. The structural details of amyloid fibrils and protofibrils, and the pathways by which these species form have not been completely elucidated, and OMPs could serve as additional model proteins for these studies. We previously demonstrated that U_AQ_ OmpA oligomerization follows a nucleated growth polymerization model with a critical nucleus of three molecules [7], and further kinetic analysis could reveal additional information about fibril formation. For example, the kinetic parameters governing the growth of the nucleus have not been elucidated, and it is unclear from our data whether additional on-pathway or off-pathway intermediate sized species are formed either stably or transiently during fibril formation.

Another area of interest is how amino acid sequence contributes to amyloid fibril formation. It is perhaps not too surprising that OmpA forms fibrils, as it has been shown that polypeptide sequences with a high propensity to form β-sheets, especially with alternating hydrophobic and hydrophilic residues (as are found in OMP β-barrels) are more prone to form amyloid fibrils [24]. However, we previously showed that OMPs have largely varying propensities to oligomerize, even though they all possess the same general pattern of hydrophobic/hydrophilic residues in their transmembrane segments [6]. This raises the question of how the exact OMP sequence governs self-association, as well as (hypothesized) fibril formation, thus motivating compelling new areas of research.

Amyloid fibril formation by OMPs also has important biological implications. Unfolded OMPs must traverse the aqueous periplasmic space before folding into the outer membrane, so the cell must have developed mechanisms to prevent the formation of large insoluble fibrils, which would be highly detrimental to cellular function. It is thought that soluble chaperone proteins, such as Skp and SurA, bind to OMPs and prevent self-association during transport across the periplasm, but the exact mechanism by which this occurs is unclear [25, 26]. Therefore, studying how chaperones bind OMPs and prevent oligomerization, and subsequent fibrillization, could be useful for designing treatments to inhibit amyloid fibril (and/or protofibril) formation in the context of neurodegenerative diseases.
